# Experimental and Simulation Study of Proton Exchange Membrane Fuel Cell with 12 µm Thick Membrane over the Temperature Range of 80 °C to 120 °C

**DOI:** 10.3390/membranes15030072

**Published:** 2025-03-01

**Authors:** Yunfei Zhang, Zhengrui Xiao, Xiaoyang Zhao, Jian Wang, Yadong Wang, Jun Yu

**Affiliations:** 1 State Key Laboratory of Advanced Technology for Materials Synthesis and Processing, Wuhan University of Technology, Wuhan 430070, Chinaywang@whut.edu.cn (Y.W.); 2 Hubei Key Laboratory of Fuel Cells, Wuhan 430070, China

**Keywords:** PEMFC, increasing temperature, electrochemical impedance, self-humidification, 3D model

## Abstract

Recent advancements have been made in understanding the mechanisms and perspectives of fuel cells operating at elevated temperatures. However, the changes in electrochemical processes within the membrane electrode assembly remain unclear. This study aims to investigate the performance variation laws of membrane electrode assemblies composed of Gore12 during operation at an increasing temperature ranging from 80 to 120 °C, utilizing overpotential decomposition and electrochemical impedance analysis. The experimental results indicate that increasing back pressure can improve the performance of fuel cells, particularly at higher temperatures. The charge transfer resistance initially decreases and then increases with temperature. Furthermore, combined with the simulation results, it is demonstrated that Gore12’s thin membrane structure provides excellent self-humidification, which ensures efficient proton conduction at low relative humidity. These findings offer new insights into improving the performance of PEMFCs and enabling stable operation at high temperatures.

## 1. Introduction

A proton exchange membrane fuel cell (PEMFC) is an energy conversion device that can convert hydrogen energy into electric energy. PEMFCs offer advantages such as high energy conversion efficiency, fast start-up speed, environmental friendliness, and system simplicity. They have been researched and applied in various fields, including transportation, aviation, military equipment, and power supply systems [1]. To meet commercialization demands, researchers are focused on enhancing the power density of PEMFCs, reducing costs, and improving reliability and durability. Studies have shown that operating PEMFCs at high temperatures above 90 °C improves reaction kinetics and catalyst efficiency effectively, enhances catalyst tolerance to carbon monoxide (CO), and simplifies water and thermal management [2,3]. However, excessively high operating temperatures increase system complexity and cost. Both low-temperature and high-temperature operation conditions pose limitations to the application of PEMFCs. In 2020, Advent Technologies signed development agreements with Los Alamos National Laboratory (LANL), Brookhaven National Laboratory (BNL), and the National Renewable Energy Laboratory, aiming to develop efficient PEMFCs capable of operating between 80 °C and 150 °C. Designing a PEMFC that can operate flexibly and stably in the temperature range between low and high temperatures and in diverse environments is of great significance in promoting the widespread application of fuel cell technology in more fields.

As a key component of PEMFCs, the proton exchange membrane (PEM) conducts protons and effectively isolates the anode and cathode gas reactants. Traditional Nafion membranes have been extensively developed and have dominated the PEMFC field. However, when the temperature of the cell with a Nafion membrane reaches above 100 °C, significant ohmic loss occurs due to the reduced water content, which seriously diminishes performance [4,5]. Passos et al. [6] evaluated the effects of different types of Nafion membranes (from 175 µm to 50 µm) on cell performance, indicating that cells with thinner membranes exhibited superior electrochemical performance, lower ohmic resistance, and improved oxygen mass transfer. Consequently, developing ultra-thin PEMs to achieve low ohmic resistance at high temperatures and low humidity has become a critical research direction [7]. Currently, the minimum thickness of pure perfluorinated sulfonic acid membranes is 25 µm. To further minimize the thickness of PEMs while ensuring good mechanical properties, the company Gore has developed a reinforced PEM by combining a Nafion membrane with an expanded polytetrafluoroethylene membrane [8]. This combination offers excellent chemical stability and mechanical properties, enabling thickness reductions to 8 µm and lowering costs by using less Nafion solution, which supports commercialization. Kienitz et al. [9] evaluated the performance of PEMs ranging from 10 µm to 35 µm in thickness and found that under dry conditions at 95 °C, membranes of 10 µm and 35 µm exhibited 2000 mA·cm^−2^ and 750 mA·cm^−2^ at 0.2 V, respectively, and the latter’s ohmic resistance was about 1.5 times that of the former. In addition, Lou et al. [10] compared Gore12 and Nafion117’s physicochemical properties from 70 °C to 90 °C and found that the water absorption rate of Gore12 increased more significantly with increasing temperature, and the dependence on water activity was lower. To fully understand the high-temperature characteristics of membrane electrode assembly (MEA) with ultra-thin membranes, Yin et al. [11] comprehensively analyzed Gore12-based PEMFCs in the temperature range from 80 °C to 95 °C, and the results showed that the ohmic loss increased with the rising temperature. While charge transfer resistance and oxygen mass transfer resistance decreased, the performance of cells at a high current density is improved with increasing temperature. Additionally, thinner membranes can enhance catalyst utilization [12], promoting the development of PEMFCs with a low platinum load. Toyota has applied ultra-thin membrane technology in Mirai vehicles, with the third generation of the Mirai operating efficiently and stably at 105 °C [13], which emphasizes the importance of ultra-thin membrane research at high temperatures.

Due to the complex structure of PEMFCs and the cross-influencing factors, experiments alone cannot fully elucidate performance changes, and more experiments and modeling are needed. The simulation of PEMFCs is generally categorized into microscale models based on molecular dynamics and macro-models based on semi-empirical equations [14,15]. Jiao and Li et al. [16,17] first developed a three-dimensional HT-PEMFC model that accounts for thermal, substance transport phenomena, and electrochemical dynamics. They simulated the effects of various parameters, such as temperature, humidity, and back pressure, on PEMFC performance, and the simulation results were consistent with the experimental data, validating the model as a reliable tool.

Although the performance of ultra-thin membrane fuel cells is focused on by numerous studies, systematic high-temperature testing (>100 °C) is limited. In this study, MEAs with Gore12 were prepared to analyze the dynamic changes in the electrochemical impedance and cell output performance under various back pressures between 80 °C and 120 °C, and the overpotential was decomposed to accurately identify the contributions of each polarization component. Based on this, the differential impact mechanisms of different pressures on the PEMFC performance with increasing temperature were discussed, aiming to reveal the key factors for cells to achieve stable and high outputs at high temperatures. Additionally, the self-humidification abilities of Gore12 and traditional Nafion212 were compared, which is critical to fully explore ultra-thin PEM’s potential application. Furthermore, a three-dimensional steady-state numerical model of PEMFCs was constructed using computer fluid dynamics (CFD) simulation technology to simulate and analyze PEMFCs, taking into account factors related to substance transport phenomena and electrochemical dynamics, providing a systematic research basis for further understanding the characteristics of the PEMFC as the temperature increases and optimizing its performance.

## 2. Materials and Methods

### 2.1. Preparation of MEA

Initially, 0.27 g of Pt/C powder (60 wt.%, JM) was weighed and mixed with 1.4 g of deionized water to ensure thorough wetting of the powder. After ultrasonic dispersion, 2.58 g of a 5 wt.% Nafion solution (EW1100) and 0.66 g of isopropyl alcohol were added to the dispersion, and the mixture was then ultrasonically dispersed for 2 h to achieve a homogeneous catalyst slurry. This prepared catalyst paste was evenly coated on a poly-tetrafluoroethylene (PTFE) surface using the doctor-blade method and subsequently dried in an oven. A 5 cm × 5 cm catalytic-layer PTFE substrate was placed on both sides of the PEM. The assembly was then transferred to a hot press, where 100 MPa of pressure and 130 °C were applied for 90 s to form the catalytic-coated membrane (CCM). The prepared CCM was sandwiched between two gas diffusion layers (SGL-22BB, SGL Group, Wiesbaden, Germany) to form the MEA for single-cell testing. The PEMs used in the experiment were 12 µm thick Gore12 (M788.12, Gore, Newark, USA) and 50 µm thick Nafion212 (NR212, DuPont, Wilmington, USA). The platinum loading was 0.1 mg·cm^−2^ on the anode catalyst layer and 0.4 mg·cm^−2^ on the cathode catalyst layer, with a total active area of 25 cm^2^.

### 2.2. Polarization Curve

Activation of the MEA is required before testing the polarization curve to activate the active sites on the catalyst layer (CL). This procedure was conducted under H_2_/air at 250 kPa (absolute pressure (abs)). The flow rates for the anode and cathode were set at 1325 mL·min^−1^ and 3000 mL·min^−1^, respectively, maintaining a constant voltage of 0.5 V for 3h until the current stabilized.

For the polarization curve test, current density intervals of 50–100 mA·cm^−2^ were used. At each current density, the corresponding stable voltage was recorded to obtain the I-V curve, and each current was held for 1 min. The test atmosphere was H_2_/air, with anode and cathode flow rates of 1325 mL·min^−1^ and 3000 mL·min^−1^. The test temperatures were controlled at 80 °C, 100 °C, and 120 °C, with back pressures set at 200 kPa, 250 kPa, and 300 kPa (abs). After the measurement at 120 °C, the polarization curve test at 80 °C was repeated to assess MEA degradation, and the maximum change was within 0.01 V, indicating that the cell could be successfully tested at high temperatures. The polarization fitting processing of the polarization curves is shown in the Supplementary Information.

### 2.3. Electrochemical Impedance Spectroscopy

Electrochemical impedance spectroscopy (EIS) is one of the most important characterization methods for PEMFCs [18], which can reflect the microscopic characteristics of electrochemical systems, and is helpful for optimizing the structure of fuel cells and selecting the most suitable operating conditions [19].

In this study, the in situ EIS technique was employed to evaluate the performance of the MEA under actual operating conditions. The test conditions were consistent with the polarization curve test, and using galvanostatic conditions, the frequency range was 10 kHz–0.01 Hz, with an amplitude set at 10% of the applied load. Each load condition was tested at least three times to ensure reliability, and the resistance values reported are the averages of these tests.

**Figure 1:**
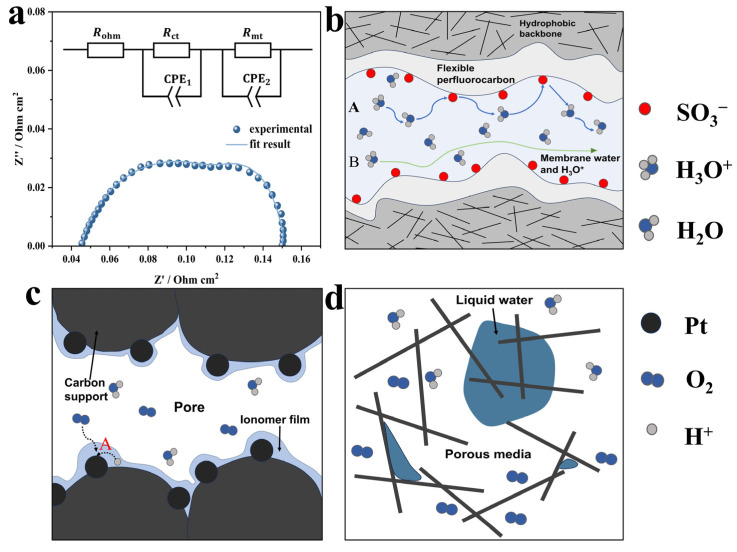
(**a**) EIS of PEMFC and equivalent circuit, (**b**) proton conduction mechanism, (**c**) three-phase reaction interface of cathode CL, and (**d**) oxygen transport in porous media.

The Nyquist plot obtained from EIS and the corresponding equivalent circuit are shown in Figure 1a. The Nyquist plot generally consists of two semi-circular arcs. The *x*-axis represents the real axis, while the *y*-axis represents the imaginary axis. The left side of the real axis is the high-frequency region, and the intersection of the impedance arc with the *x*-axis in this region indicates the ohmic resistance $R_{ohm}$ [20], which is primarily governed by membrane resistance. Proton transport within the membrane generally occurs via the Grotthuss mechanism and vehicular mechanism [21,22], as illustrated in Figure 1b. In general, both mechanisms occur simultaneously, and the vehicular mechanism is more susceptible to water content. The first semi-circular in the high-frequency region represents charge transfer resistance $R_{ct}$, which reflects the difficulty of electron transfer from the electrode surface to the catalyst surface or from adsorbed substances to the electrode. $R_{ct}$ is influenced by catalyst activity, reactant concentration, and proton resistance at the active sites of the three-phase reaction interface (Figure 1c(A)), and it provides insight into the electrochemical reaction kinetics of the MEA [23]. The second semicircle represents the mass transport resistance $R_{mt}$ in porous materials (Figure 1d) and flow channels, which appears on the cathode side where water is generated.

### 2.4. Cyclic Voltammetry Curve

H_2_ and N_2_ were introduced into the anode and cathode at gas flow rates of 1325 mL∙min^−1^ and 3000 mL∙min^−1^, respectively. Before the testing was performed, the cell was purged under these gas flow conditions until the voltage decreased to 0.1 V, and the voltage was maintained for 30 min. Cyclic voltammetry (CV) was performed over a range of 0.05 V to 1.2 V for five cycles at a scan rate of 20 mV∙s^−1^.

## 3. Model Descriptions

**Figure 2:**
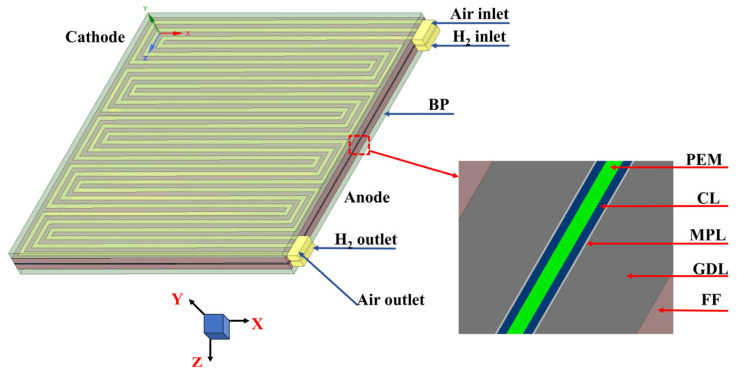
Schematic of PEMFC model.

Figure 2 shows the geometric domain of the simulation calculation. The flow field of the PEMFC applied in this study is a serpentine flow channel, and the three-dimensional domain consists of 11 components, including the bipolar plates (BPs), flow fields (FFs), gas diffusion layers (GDLs), microporous layers (MPLs), CLs on both the anode and cathode sides, and a PEM. In the meshing rules, each component is divided into 284,050 grids, and the entire computing domain is divided into 3,129,550 grids.

### 3.1. Model Hypothesis

1. The PEMFC system operates in a stable state;
2. The PEM is considered to be a solid material that does not permeate gases;
3. The flow mode of reactant gas in the flow channel is laminar flow due to the low Reynolds number;
4. The reaction gases behave ideally;
5. The GDL, CL, and PEM are considered to be homogeneous porous media.

### 3.2. Governing Equations

The governing equations in the 3D steady-state model are as follows, with $S_{m}$, $S_{u}$, $S_{i}$, $S_{T}$, $S_{ele}$, and $S_{pro}$ as the corresponding quality source items [17]:

Mass conservation equation:

(1) $$\nabla \cdot (\varepsilon \rho \vec{u})=S_{m}$$

*ε* represents the porosity of porous materials (the GDL, MPL, and CL), ρ is the density of the mixed gas (kg·m^−3^), and $\vec{u}$ is the velocity vector (m·s^−1^).

Momentum conservation equation:

(2) $$\nabla \cdot \left(\varepsilon \rho \vec{u}\vec{u}\right)=-\varepsilon \nabla p+\nabla \cdot \left(\varepsilon \mu \nabla \vec{u}\right)+S_{u}$$

p is the pressure (Pa), and μ is the viscosity.

Species conservation equation:

(3) $$\nabla \cdot (\varepsilon \vec{u}c_{i})=\nabla \cdot {\vec{J}}_{i}+S_{i}$$

$c_{i}$ is the molar fraction of the substance, and ${\vec{J}}_{i}$ represents the diffusive mass flux vector (mol·m^−2^·s^−1^).

Energy conservation equation:

(4) $$\nabla \cdot (\varepsilon \rho C_{p}\vec{u}T)=\nabla \cdot (\lambda^{eff}\nabla T)+S_{T}$$

$C_{p}$ is the specific heat capacity of the gas mixture (J·mol^−1^·K^−1^), and $\lambda^{eff}$ is the effective thermal conductivity (W·m^−1^·K^−1^).

Charge conservation equation:

(5) $$\nabla \cdot \left(k_{e}\nabla \varphi_{ele}\right)+S_{ele}=0$$

(6) $$\nabla \cdot \left(k_{m}\nabla \varphi_{pro}\right)+S_{pro}=0$$

$k_{e}$ and $k_{m}$ represent the electronic conductivity (S·m^−1^) of the electrode and the proton conductivity of the membrane, respectively. The solid phase potential $\varphi_{ele}$ (V) represents the transport of electrons through the electrode solid conducting material, and the membrane phase potential $\varphi_{pro}$ (V) represents the transport of protons through the membrane [24].

### 3.3. Water Transport in PEM

Water in the membrane in the form of membrane water serves as the medium of proton transport. The membrane water transfer equation (Equation (7)) was established by Springer et al. [25] based on the diffusion theoretical model, which mainly includes electro-osmotic drag (EOD) [26], diffusion, and the pressure differential diffusion of water in the membrane:

(7) $$\varphi_{w}=2.5\frac{\lambda }{22}\frac{i}{F}+D_{w}c_{s}\frac{\partial \lambda }{\partial y}+\frac{\kappa_{p}}{\mu }c_{w}\frac{dp}{dy}$$

λ is the water content, representing the number of water molecules contained in each sulfonic acid group in the proton exchange membrane; $c_{s}$ is the concentration of sulfonic acid groups (mol·m^−3^); $D_{w}$ is the diffusion coefficient of water in the membrane (m^2^·s^−1^); $\kappa_{p}$ is the hydraulic permeability coefficient within the membrane; and $c_{w}$ is the concentration of water in the membrane (mol·m^−3^).

### 3.4. Boundary Conditions

1. The anode/cathode inlet is set as the mass inlet, and the mass flux and mass fraction are set according to the working condition;
2. The outlet of the anode and cathode adopts pressure boundary conditions to reflect the emission characteristics of the actual fuel cell;
3. The wall is set as a constant temperature wall to simulate the thermal stability of the cell during operation;
4. The simulation results use current to calculate voltage, set the anode potential to 0 V, and set the cathode according to the current density.

Some of the parameters mentioned above that need to be input into the model are shown in Table S1. The CFD software FLUENT 19.2 was used to solve the control equation based on the double-precision format of the finite volume method. In order to ensure the stability of numerical calculation and achieve convergence, this study applied appropriate relaxation factors to each control variable, and all residuals were less than 10^−6^. As shown in Figure S1, the experimental data fit well with the simulation data, which verifies the accuracy of the simulation data.

## 4. Results and Discussion

### 4.1. Electrochemical Performance of PEMFC

**Figure 3:**
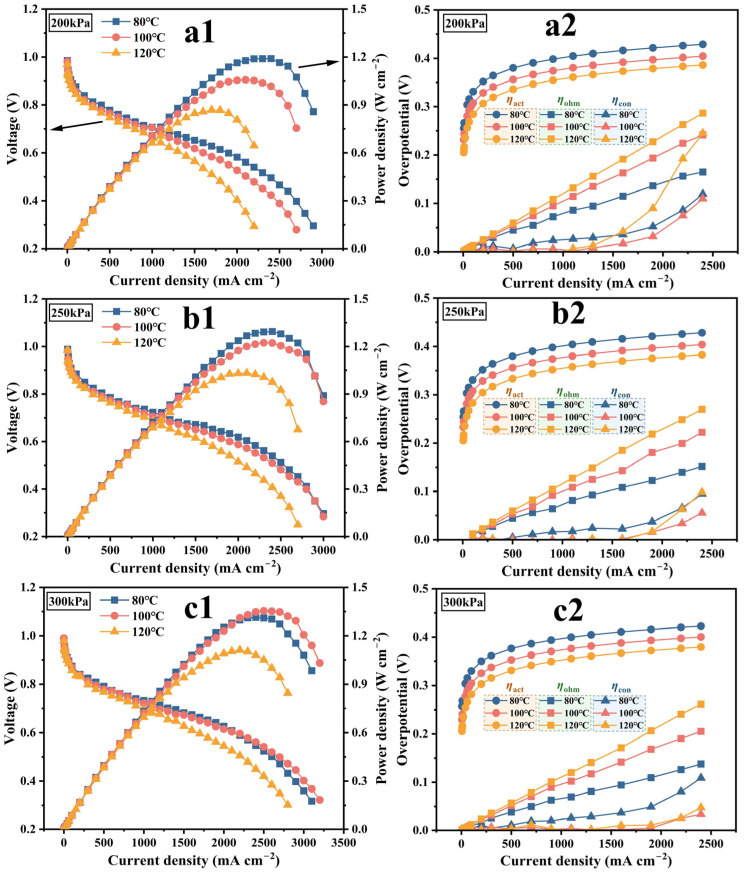
Under the conditions of 43% RH, 80/100/120 °C, and 200/250/300 kPa back pressures, (**a1**–**c1**) the polarization curves and (**a2**–**c2**) corresponding overpotential decomposition results of a single cell composed of Gore12.

The polarization and power density curves of the PEMFC composed of Gore12, operating from 80 °C to 120 °C at 43% relative humidity (RH), are depicted in Figure 3a1–c1. It is obvious that back pressure significantly influences the performance of the PEMFC. As the back pressure increases, the performance difference between adjacent temperatures gradually decreases. Especially at 300 kPa, the performance at 80 °C and 100 °C intersect. At constant temperatures, the performance improves with higher back pressure, especially at elevated temperatures. At a current density of 2000 mA·cm^−2^, when the back pressure is increased from 200 kPa to 300 kPa, the performance of the PEMFC at 80 °C, 100 °C, and 120 °C is increased by 0.045 V, 0.088 V, and 0.142 V, respectively. To further analyze the internal root causes of the performance differences of the PEMFC operating under various conditions, polarization curves were decomposed according to Equations (S2)–(S9), as depicted in Figure 3a2–c2.

First, the cell exhibits significant activation overpotential ($\eta_{act}$) across the current density range, which largely depends on the intrinsic properties of the catalyst caused by the reaction kinetics. Increasing the temperature can reduce the contribution of $\eta_{act}$ to the cell. Ohmic overpotential ($\eta_{ohm}$) is dominated by proton conduction in the PEM. Although an elevated temperature can enhance the proton diffusion coefficient [27] and water diffusion coefficient, most of the water within the MEA exists as vaporous water and is easily expelled with the gas flow. Additionally, elevated temperatures promote EOD, leading to a more rapid dragging of water from the anode to the cathode, which accelerates PEM dehydration [28]. Therefore, a reduction in water content decreases the proton carrier in the form of hydrogen–water ions (e.g., H_3_O^+^ and H_5_O_2_^+^) and disrupts the continuous hydrogen bond network structure, eventually leading to an increase in $\eta_{ohm}$ as the temperature rises from 80 °C to 120 °C. Due to the linear relationship between $\eta_{ohm}$ and current density, its contribution to voltage loss grows with increasing current density. This effect is particularly evident at a high current density, where the difference in $\eta_{ohm}$ between 80 °C and 120 °C becomes most apparent. This could be attributed to the fact that EOD is especially prominent at a high current density [29], which exacerbates membrane dehydration caused by the temperature increase under such conditions. Consequently, many studies focus on reducing the ohmic loss of membranes at low humidity [30,31].

As the current density increases to a relatively high level, such as above 1500 mA∙cm^−2^, the electrode reaction accelerates. On the one hand, the consumption of the reaction gases at the three-phase reaction interface increases; on the other hand, increased water production on the cathode side tends to block gas transport channels. These factors significantly reduce the concentrations of reactant gas reaching the active sites of catalysts, sharply elevating the concentration overpotential ($\eta_{con}$) at high current densities, which critically impacts the high-power output of the PEMFC. Generally, it is believed that appropriately increasing the temperature can reduce the liquid water content and accelerate the diffusion rate of reactants, thereby reducing the mass transport resistance and creating more favorable mass transport conditions for the electrochemical reactions [32]. However, elevated temperatures also lower oxygen partial pressures, as shown in Table S2. For example, at a 200 kPa back pressure, the issue is particularly prominent when the operating temperature rises to 120 °C, which is caused by the tradeoff between the gas diffusion and solubility in the CL. According to Henry’s law, oxygen solubility on the catalyst is proportional to its partial pressure, and the oxygen mole fraction is only 11% under this condition. The effect of decreasing the solubility of the reaction gas may be greater than that of increasing the diffusivity of the gas [33,34], resulting in a significant increase in $\eta_{con}$ at 120 °C. In general, as the temperature increases at 200 kPa, the cell performance decreases significantly due to ohmic loss and concentration loss, as shown in Figure 3a1. With the increase in the back pressure to 250 kPa, the $\eta_{con}$ under high-temperature operating conditions decreases with the increase in the oxygen partial pressure, and the cell performance at each temperature is improved, and the performance difference between 80 °C and 120 °C is gradually reduced. When the back pressure is further increased to 300 kPa, the maximum $\eta_{con}$ appears at 80 °C, which is because the solubility of oxygen in the CL increases with the rise of reactant partial pressure, thus accelerating the rate of cathode oxygen reduction to produce liquid water. At this point, the increasing temperature shows the advantage of reducing liquid water and promoting the uniform distribution of oxygen in the CL [34], which is most obvious at a high current density, as seen in Figure 3c2 where $\eta_{con}$ drops significantly at 100 °C and 120 °C. Therefore, when increasing from 80 °C to 100 °C at 300 kPa, the decrease in $\eta_{con}$ and $\eta_{act}$ is greater than the increase in $\eta_{ohm}$. As a result, the performance of the cell at 100 °C is higher than that at 80 °C at a high current density, and the peak power density also increases by 0.033 W·cm^−2^. However, when the temperature rises to 120 °C, the performance of the cell decreases due to a higher $\eta_{ohm}$ and insufficient reactant concentration. The increase in back pressure can not only effectively alleviate the performance degradation caused by increasing temperature, for example, when working at 2000 mA·cm^−2^, the voltage difference between 80 °C and 120 °C decreases from 0.179 V at 200 kPa to 0.081 V at 300 kPa, but also reveals that the decrease in reactant concentration at high temperatures is one of the important factors causing the deterioration of cell performance.

**Figure 4:**
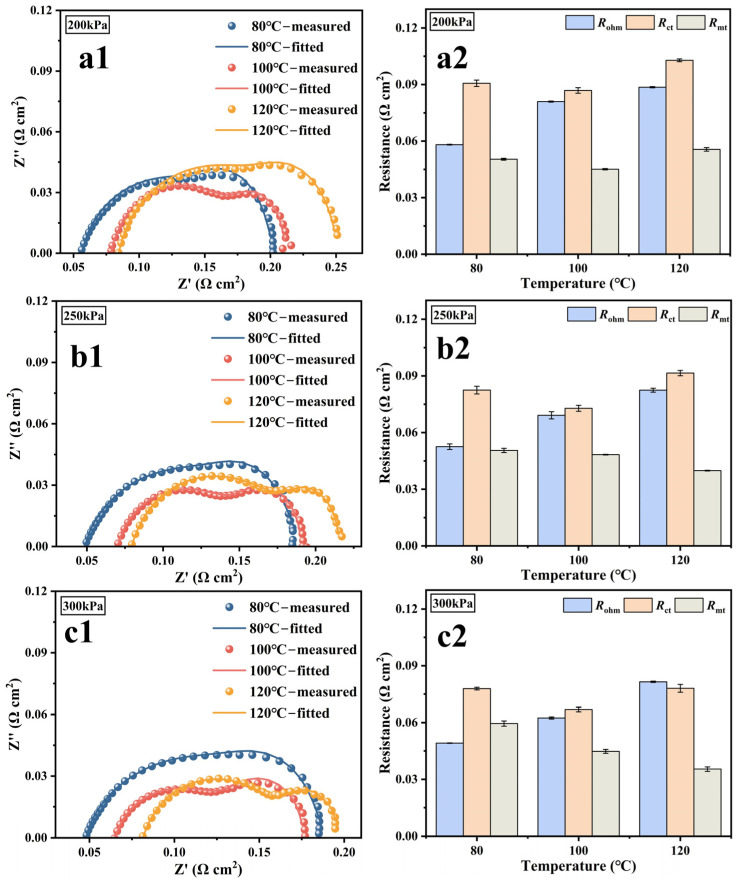
Under the conditions of 43% RH, 80/100/120 °C, and 200/250/300 kPa back pressures, respectively, the (**a1**–**c1**) EIS and their fitted curves and (**a2**–**c2**) the area resistance values of the $R_{ohm}$, $R_{ct}$, and $R_{mt}$ at 0.65 V.

Figure 4a1–c1,a2–c2 present EIS and the corresponding fitting results. $R_{ohm}$ clearly increases with rising temperature, consistent with the change in $\eta_{ohm}$ in Figure 3. $R_{ct}$ decreases as the temperature rises from 80 °C to 100 °C, indicating that the electrochemical reaction kinetics of the PEMFC with the ultra-thin membrane Gore12 accelerate with increasing temperature. And this decreasing trend becomes more pronounced with the increase in back pressure. $R_{ct}$ decreases 0.004 Ω·cm^2^ at 200 kPa, 0.01 Ω·cm^2^ at 250 kPa, and 0.013 Ω·cm^2^ at 300 kPa, respectively, which further underscores the importance of the oxygen concentration in high-temperature conditions. When the temperature further increases to 120 °C, $R_{ct}$ rises, which is mainly caused by a higher $R_{ohm}$ and lower oxygen concentration. The change laws of $R_{ct}$ and $\eta_{act}$ are inconsistent, as the calculation of $\eta_{act}$ is not solely based on pure kinetic considerations. According to the Nernst equation, the dynamic change in gas partial pressure causes the reversible potential to shift under a given humidity and total pressure condition. As shown in Table S2, at 43% RH, when the back pressure is between 200 kPa and 300 kPa, and the temperature rises from 80 °C to 120 °C, the reversible potential decreases by about 0.06 V.

Both $R_{ohm}$ and $R_{ct}$ decrease with the increase in pressure, as shown in Figure 4. The underlying mechanism is as follows: increasing reactant pressure increases the concentration of protons and oxygen participating in the reaction at the three-phase interface. According to the reaction kinetics process, more substances participate in the reaction, thus effectively reducing $R_{ct}$. The accelerated cathode oxygen reaction results in more water being produced, driven by the concentration gradient, water easily diffuses from the cathode to the anode, and the water content in the membrane increases, making $R_{ohm}$ decrease with the increase in back pressure. This process of effectively using the water generated during the cell operation to maintain its own good proton conductivity is called self-humidification of the membrane [35]. Under the operating condition of 200 kPa back pressure and 120 °C, the performance of both $R_{mt}$ and $\eta_{con}$ confirm that oxygen transport is significantly hindered at low reactant concentrations and high temperatures. Applying back pressure at elevated temperatures is more beneficial in reducing $R_{mt}$ than at low temperatures.

**Figure 5:**
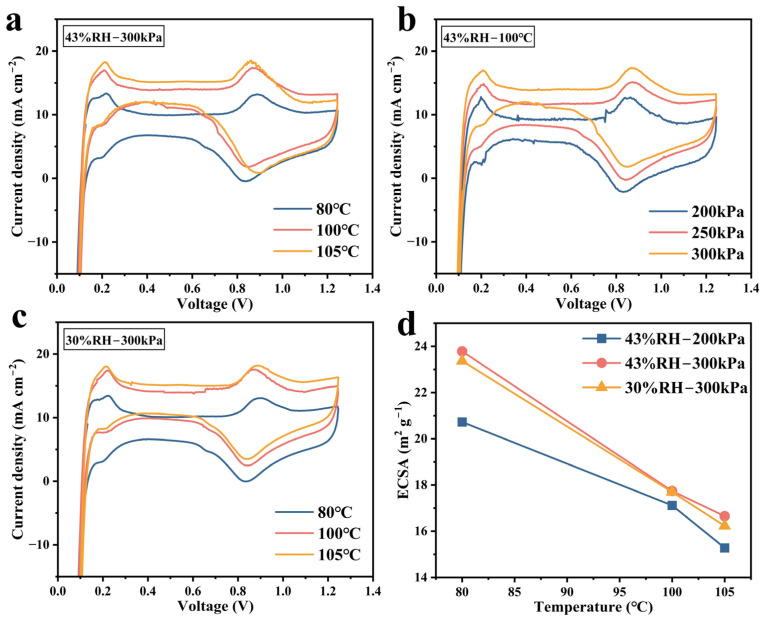
The CV curves at 1325 mL∙min^−1^ pure H_2_ on the anode and 3000 mL∙min^−1^ N_2_ on the cathode under different operating conditions: (**a**) 300 kPa and 43% RH at 80/100/105 °C; (**b**) 100 °C and 43% RH at 200/250/300 kPa back pressures; and (**c**) 300 kPa and 30% RH at 85/100/105 °C. (**d**) ECSA calculated values.

Figure 5 shows CV curves under different operating conditions. The CV curves are upwardly elevated with the increase in temperature, as shown in Figure 5a,b, indicating that under the same excitation voltage, the response current increases with the increase in temperature and back pressure, which is caused by the acceleration of the electrochemical reaction rate [11]. However, ECSA is calculated from Equation (S1), as shown in Figure 5d, which indicates that the increase in temperature leads to a significant reduction in ECSA. This decrease occurs because the higher temperature inhibits hydrogen adsorption, reducing the total charge absorbed, and diminishes the Pt/water interface. It can be observed that the trend of ECSA with temperature is inconsistent with that of $R_{ct}$ mentioned above. $R_{ct}$ is influenced by both the electrochemical surface area and the concentration of reactants, and it reflects the reaction kinetics of the MEA under specific conditions. However, ECSA merely represents a physical quantity of the catalyst’s active surface area. Therefore, it is inadvisable to rely solely on ECSA to evaluate the reaction kinetics of PEMFCs at different temperatures.

### 4.2. Self-Humidification of the Membrane

**Figure 6:**
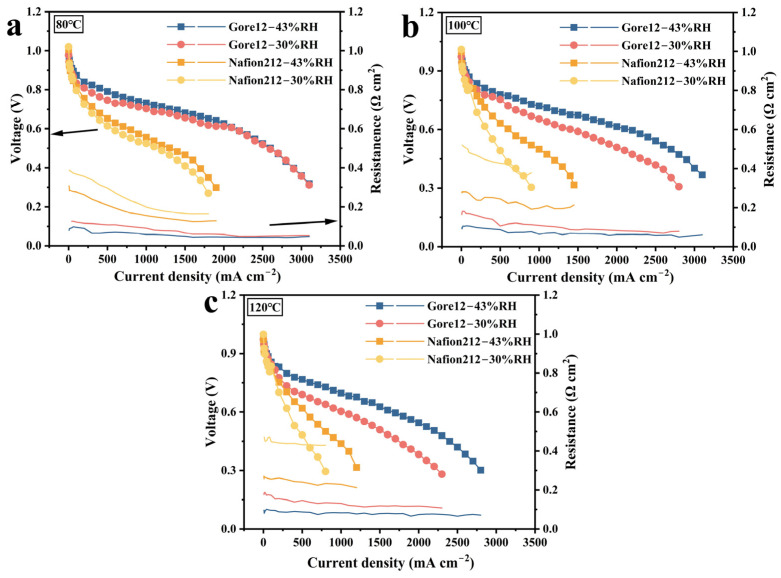
At 300 kPa back pressure, polarization and HFR curves of cells composed of Gore12 and Nafion212 under 30%/43% RH and temperatures of (**a**) 80 °C, (**b**) 100 °C, and (**c**) 120 °C.

In the PEMFC system, effective water management is crucial for maintaining the performance of the cell. As a key characteristic, self-humidification relies on the combined action of EOD and back-diffusion, which ensures a proper distribution of water molecules within the cell. The polarization curves and high-frequency resistance (HFR) (which is automatically recorded during the testing of the polarization curve and represents ohmic resistance at 1 kHz) [36] at different humidities were measured to further study the self-humidification effect of different membranes with increasing temperature, as shown in Figure 6. Due to its longer proton transport path and higher ohmic impedance, Nafion212 exhibits significantly lower cell performance compared to Gore12.

Clearly, at 300 kPa and 80 °C, the polarization curves of the Gore12-based PEMFC at 43%/30% RH gradually converge and cross at a specific point with the increase in the current density, and its HFR also gradually converges. This can be attributed to the insufficient water content in the PEM at lower current densities under 30% RH, resulting in inferior performance compared to under 43% RH. At higher current densities, the increased water content in the cathode side of the membrane can lead to flooding in the MEA with higher relative humidity, thereby accelerating the decline in performance. Similarly, the HFR and I-V curves of the MEA composed of Nafion212 converge with the increase in current density, but the effect is not as obvious as that of Gore12. Additionally, the variation in HFR difference between 43% RH and 30% RH exhibits an upward trend as the temperature rises. For example, at 2000 mA·cm^−2^, the resistance difference between 43%/30% RH for the Gore12-based cell is measured to be 0.013 Ω·cm^2^ at 80 °C, 0.021 Ω·cm^2^ at 100 °C, and 0.051 Ω·cm^2^ at 120 °C, indicating that the difficulty of the cell self-humidifying is significantly increased at higher temperatures [37]. Compared with Gore12, the influence of humidity change on the performance of the Nafion212-based PEMFC is more obvious because the thicker membrane makes the diffusion of water to the anode more difficult. As shown in Figure 6b, when the temperature is kept at 100 °C and the humidity is reduced from 43% to 30%, the HFR of the Nafion212-based cell at a high current density increases by about 70% while that of the Gore12-based cell only increases by about 30%.

In short, the self-humidification ability in the thinner membrane is stronger, and it is more difficult for a PEMFC to achieve self-humidification at higher temperatures.

### 4.3. Simulation Results

Due to the complex structure of a PEMFC, it is challenging to intuitively understand the actual dynamic changes and transport processes of substances (water, oxygen, etc.) in the MEA, and the experimental results can only be inferred and interpreted based on theories. Therefore, simulation research serves as an effective approach. All simulations in this paper depict the substance distribution of 1/2 of the corresponding structure in the thickness direction.

**Figure 7:**
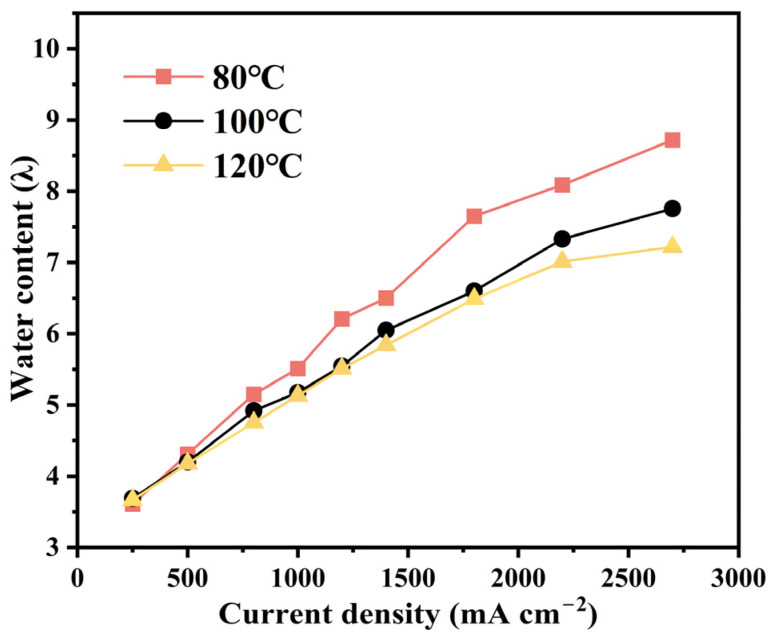
At 200 kPa and 43% RH, the water content in PEM with the increase in current density during operation at 80/100/120 °C.

Figure 7 illustrates the change in water content within a 12 µm thick membrane, and the data indicate that the water content increases with the rising current density. This is because the acceleration of the cathode reaction enhances water production and increases the water flux diffusing to the anode, which also proves the membrane’s self-humidification capability. As the temperature rises, the conversion rate of liquid water to vapor increases, resulting in a decrease in the overall water content of the PEM. At high current densities, temperature has the most obvious effect on water content, and the variation in water content at different temperatures correlates with the difference in ohmic overpotential, as shown in Figure 3a2–c2.

**Figure 8:**
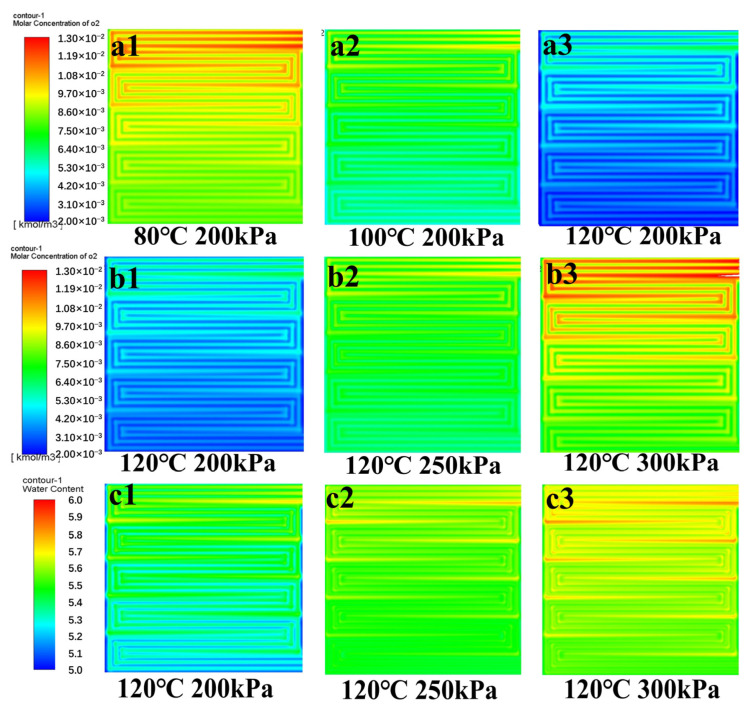
At 1200mA·cm^−2^, (**a1**–**a3**) the distribution of oxygen molar concentration in the cathode CL at 200 kPa back pressure and 80/100/120 °C, (**b1**–**b3**) the distribution of oxygen molar concentration in the cathode CL at 120 °C and 200/250/300 kPa back pressures, and (**c1**–**c3**) the distribution of water content in PEM at 120 °C and 200/250/300 kPa back pressures.

Moreover, the distribution of the molar concentration of oxygen in the cathode CL is depicted in Figure 8a1–a3. It can be seen that as the temperature rises to 100 °C, the oxygen distribution in the CL becomes more uniform, enhancing catalyst utilization. However, at 120 °C, the molar concentration of oxygen in the CL decreases significantly. Increasing back pressure raises the partial pressure of the reaction gases, thereby increasing the molar concentration of oxygen, as shown in Figure 8b1–b3. This enhancement accelerates the electrochemical reaction and further improves the back diffusion ability of water, which helps to humidify the membrane, as shown in Figure 8c1–c3.

**Figure 9:**
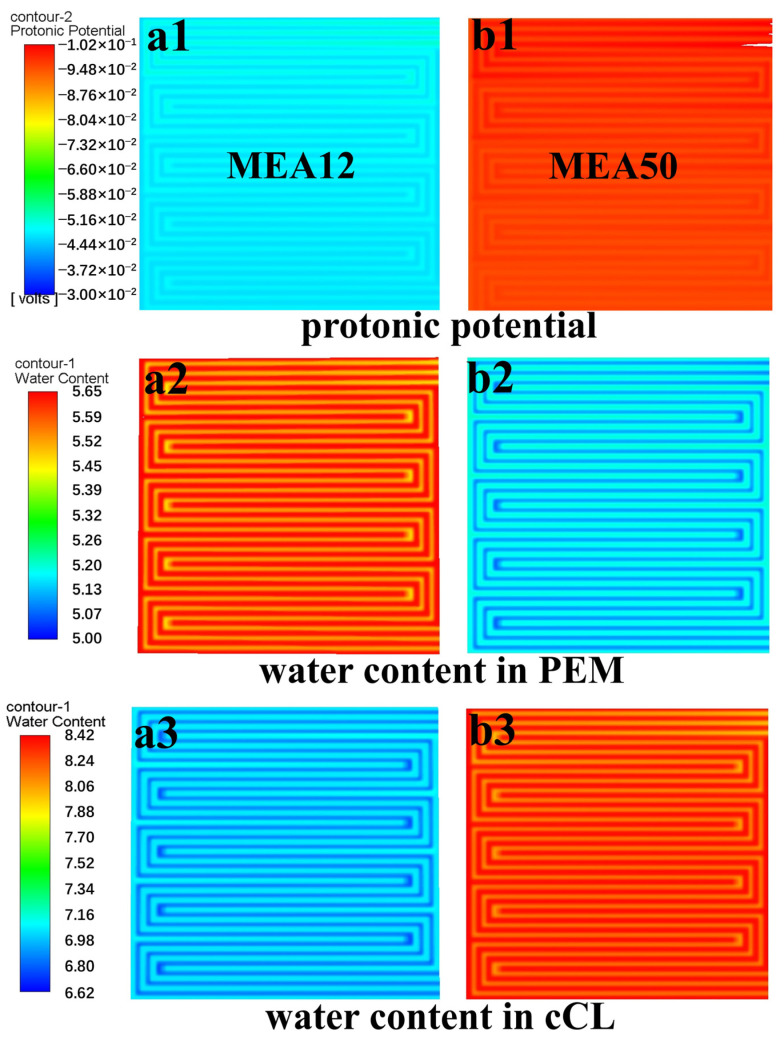
At 100 °C and 250 kPa, proton potential in membrane of (**a1**) MEA12 and (**b1**) MEA50, water content distribution in the PEM of (**a2**) MEA12 and (**b2**) MEA50, and water content distribution in cathode CL of (**a3**) MEA12 and (**b3**) MEA50.

PEMFC models with different membrane thicknesses were established. The MEA with a 12 µm thick membrane is referred to as MEA12, while the one with a 50 µm thick membrane is MEA50. Figure 9a1–a3, respectively, represent the proton potential, water content distribution in the membrane, and the cathode CL of MEA12 at a temperature of 100 °C and pressure of 250 kPa, while Figure 9b1–b3 are the corresponding simulation results for MEA50. The proton potential of the anode CL was set to 0. The greater the obstruction of the proton transport from the anode to the cathode results in increased proton potential loss and the more negative its value. As shown in Figure 9a1,b1, the proton potential value of MEA50 is significantly more negative than that of MEA12, corresponding to the water content in Figure 9a2,b2, indicating that a thinner PEM can effectively utilize reactant water and exhibit superior proton conductivity at high temperatures and low humidity. However, the reactant water in the MEA with a thicker membrane tends to accumulate on the cathode side (as shown in Figure 9a3,b3), which will occupy numerous gas transport channels and hinder oxygen transport. Thus, thinner membranes facilitate better water management and oxygen mass transfer on the cathode side.

**Figure 10:**
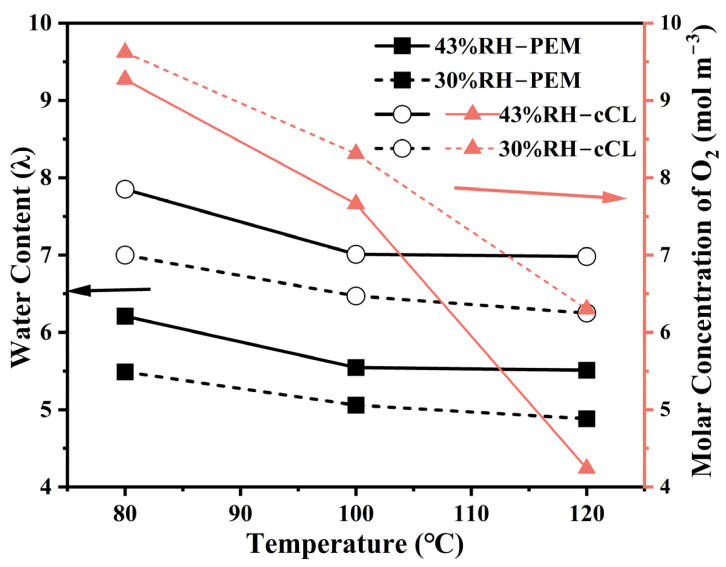
At 200 kPa, the water content and oxygen molar concentration of the MEA at 30%/43% RH and 80/100/120 °C.

The simulation results of the oxygen concentration and water content at different humidity in the temperature range of 80 °C to 120 °C are depicted in Figure 10. It is evident that both water content and oxygen concentration in the MEA decrease with increasing temperatures. Since the cathode CL is where water is generated by reactions, its water content consistently exceeds that in the membrane. However, increasing humidity reduces the concentration of the reaction gas, and at higher humidity, the decrease in oxygen concentration becomes more pronounced with rising temperatures, which will affect the electrode reaction involving oxygen. This is consistent with the theoretical calculation phenomenon shown in Table S2. Therefore, to achieve an optimal and stable performance in the temperature range of 80–120 °C, operation at excessively high humidity should be avoided. Consequently, the development of self-humidification membranes that can efficiently utilize reactant water is particularly crucial.

## 5. Conclusions

This experiment focused on the performance changes of a Gore12-based PEMFC over the temperature of 80 °C to 120 °C and explored the key influencing factors on the performance of the PEMFC by changing the MEA parameters. At the same time, the self-humidification effects of membranes with different thicknesses were examined. A 3D steady-state PEMFC model was developed to simulate the oxygen and water distribution changes in the MEA under various conditions. The main conclusions are as follows:

Increasing the back pressure at a constant temperature can improve the performance of a PEMFC. Notably, the effect of back pressure becomes more pronounced at higher temperatures. At 120 °C and 2000 mA·cm^−2^, when the back pressure is from 200 kPa to 300 kPa, the voltage can be increased by 0.142 V. This improvement is mainly due to a significant reduction in concentration loss. The charge transfer resistance initially decreases and then increases with temperature, which is the result of the combined effect of the oxygen concentration and water content. In addition, experimental and simulation studies show that Gore12 can better achieve self-regulation of water distribution, contributing to more efficient water management and oxygen transport within the PEMFC. In general, in order to enable a PEMFC to achieve good performance at high temperatures and low relative humidity, the development of an ultra-thin PEM is a favorable choice, and a reasonable increase in back pressure can compensate for the many negative effects of high temperatures on the cell to a large extent.

## Data Availability

The original contributions presented in the study are included in the article/Supplementary Materials; further inquiries can be directed to the corresponding author/s.

## Supplementary Materials

The following supplementary information can be downloaded at: https://www.mdpi.com/article/10.3390/membranes15030072/s1. Equation (S1): The formula representing ECSA; Equations (S2)–(S10): The formulas representing overpotential [1,38,39,40,41]; Table S1: Physical parameters and boundary conditions for modeling [37]; Table S2: Partial pressures and equilibrium overpotentials under different conditions; Figure S1: Polarization curve by experiments and modeling.

## References

[1] Yuan X., Wang H., Zhang J. (2008). PEM Fuel Cell Fundamentals. PEM Fuel Cell Electrocatalysts and Catalyst Layers: Fundamentals and Applications.

[2] Zhang J., Zhang H., Wu J., Zhang J. (2013). High-Temperature PEM Fuel Cells. Pem Fuel Cell Testing and Diagnosis.

[3] Xiao T., Wang R., Chang Z., Fang Z., Zhu Z., Xu C. (2020). Electrolyte membranes for intermediate temperature proton exchange membrane fuel cell. Prog. Nat. Sci. Mater. Int..

[4] Tang Y., Zhang J., Song C., Liu H., Zhang J., Wang H., Mackinnon S., Peckham T., Li J., McDermid S. (2006). Temperature Dependent Performance and In Situ AC Impedance of High-Temperature PEM Fuel Cells Using the Nafion-112 Membrane. J. Electrochem. Soc..

[5] Zhang J., Tang Y., Song C., Cheng X., Zhang J., Wang H. (2007). PEM fuel cells operated at 0% relative humidity in the temperature range of 23–120 °C. Electrochim. Acta.

[6] Passos R.R., Paganin V.A., Ticianelli E.A. (2006). Studies of the performance of PEM fuel cell cathodes with the catalyst layer directly applied on Nafion membranes. Electrochim. Acta.

[7] Zeng L., Lu X., Yuan C., Yuan W., Chen K., Guo J., Zhang X., Wang J., Liao Q., Wei Z. (2024). Self-Enhancement of Perfluorinated Sulfonic Acid Proton Exchange Membrane with Its Own Nanofibers. Adv. Mater..

[8] Nauman Javed R.M., Al-Othman A., Tawalbeh M., Olabi A.G. (2022). Recent developments in graphene and graphene oxide materials for polymer electrolyte membrane fuel cells applications. Renew. Sustain. Energy Rev..

[9] Kienitz B., Kolde J., Priester S., Baczkowski C., Crum M. (2011). Ultra-Thin Reinforced Ionomer Membranes to Meet Next Generation Fuel Cell Targets. ECS Trans..

[10] Lou J., Lu Y., Yang D., Pan X., Li B., Ming P. (2024). Experimental and model refinement of water content and membrane conductivity in reinforced composite proton exchange membranes. Int. J. Hydrogen Energy.

[11] Yin T., Chen D., Hu T., Hu S., Li R., Wei T., Li Y., Li Y., Xu X., Pei P. (2024). Experimental investigation and comprehensive analysis of performance and membrane electrode assembly parameters for proton exchange membrane fuel cell at high operating temperature. Energy Convers. Manag..

[12] Breitwieser M., Klingele M., Britton B., Holdcroft S., Zengerle R., Thiele S. (2015). Improved Pt-utilization efficiency of low Pt-loading PEM fuel cell electrodes using direct membrane deposition. Electrochem. Commun..

[13] Song J., Zhao W., Zhou L., Meng H., Wang H., Guan P., Li M., Zou Y., Feng W., Zhang M. (2023). Rational Materials and Structure Design for Improving the Performance and Durability of High Temperature Proton Exchange Membranes (HT-PEMs). Adv. Sci..

[14] Jinnouchi R. (2003). New insight into microscale transport phenomena in PEFC by quantum MD. Microscale Thermophys. Eng..

[15] Rasheed R.K.A., Liao Q., Caizhi Z., Chan S.H. (2017). A review on modelling of high temperature proton exchange membrane fuel cells (HT-PEMFCs). Int. J. Hydrogen Energy.

[16] Jiao K., Alaefour I.E., Li X. (2011). Three-dimensional non-isothermal modeling of carbon monoxide poisoning in high temperature proton exchange membrane fuel cells with phosphoric acid doped polybenzimidazole membranes. Fuel.

[17] Yin Y., Wang J., Yang X., Du Q., Fang J., Jiao K. (2014). Modeling of high temperature proton exchange membrane fuel cells with novel sulfonated polybenzimidazole membranes. Int. J. Hydrogen Energy.

[18] Niya S.M.R., Hoorfar M. (2013). Study of proton exchange membrane fuel cells using electrochemical impedance spectroscopy technique—A review. J. Power Sources.

[19] Ramani V.K., Cooper K., Fenton J.M., Russel Kunz H., Breitkopf C., Swider-Lyons K. (2017). Polymer Electrolyte Fuel Cells. Springer Handbook of Electrochemical Energy.

[20] Baudy M., Rondeau O., Jaafar A., Turpin C., Abbou S., Grignon M. (2022). Voltage Readjustment Methodology According to Pressure and Temperature Applied to a High Temperature PEM Fuel Cell. Energies.

[21] Kreuer K.D., Paddison S.J., Spohr E., Schuster M. (2004). Transport in Proton Conductors for Fuel-Cell Applications:  Simulations, Elementary Reactions, and Phenomenology. Chem. Rev..

[22] Kreuer K.D. (1996). Proton Conductivity:  Materials and Applications. Chem. Mater..

[23] Rao Y., Cai C., Tan J., Pan M. (2019). Oxygen Reduction Activity Indicator for Fuel Cell Catalysts at Rated Voltage. J. Electrochem. Soc..

[24] Al-Baghdadi M.A.R.S., Al-Janabi H.A.K.S. (2007). Numerical analysis of a proton exchange membrane fuel cell. Part 1: Model development. Proc. Inst. Mech. Eng. A..

[25] Springer T.E., Zawodzinski T.A., Gottesfeld S. (1991). Polymer Electrolyte Fuel Cell Model. J. Electrochem. Soc..

[26] Ji M., Wei Z. (2009). A Review of Water Management in Polymer Electrolyte Membrane Fuel Cells. Energies.

[27] Choi P., Jalani N.H., Datta R. (2005). Thermodynamics and Proton Transport in Nafion: II. Proton Diffusion Mechanisms and Conductivity. J. Electrochem. Soc..

[28] Luo Z., Chang Z., Zhang Y., Liu Z., Li J. (2010). Electro-osmotic drag coefficient and proton conductivity in Nafion^®^ membrane for PEMFC. Int. J. Hydrogen Energy.

[29] Sridhar P., Perumal R., Rajalakshmi N., Raja M., Dhathathreyan K.S. (2001). Humidification studies on polymer electrolyte membrane fuel cell. J. Power Sources.

[30] Ke C., Li X., Qu S., Shao Z., Yi B. (2011). Preparation and properties of Nafion/SiO_2_ composite membrane derived via in situ sol-gel reaction: Size controlling and size effects of SiO_2_ nano-particles. Polym. Adv. Technol..

[31] Qu E., Hao X., Xiao M., Han D., Huang S., Huang Z., Wang S., Meng Y. (2022). Proton exchange membranes for high temperature proton exchange membrane fuel cells: Challenges and perspectives. J. Power Sources.

[32] Nonoyama N., Okazaki S., Weber A.Z., Ikogi Y., Yoshida T. (2011). Analysis of Oxygen-Transport Diffusion Resistance in Proton-Exchange-Membrane Fuel Cells. J. Electrochem. Soc..

[33] Zhang J., Tang Y., Song C., Zhang J. (2007). Polybenzimidazole-membrane-based PEM fuel cell in the temperature range of 120–200 °C. J. Power Sources.

[34] Akitomo F., Sasabe T., Yoshida T., Naito H., Kawamura K., Hirai S. (2019). Investigation of effects of high temperature and pressure on a polymer electrolyte fuel cell with polarization analysis and X-ray imaging of liquid water. J. Power Sources.

[35] Zhao X., Xu L., Fang C., Jiang H., Li J., Ouyang M. (2018). Study on voltage clamping and self-humidification effects of pem fuel cell system with dual recirculation based on orthogonal test method. Int. J. Hydrogen Energy.

[36] Makharia R., Mathias M.F., Baker D.R. (2005). Measurement of Catalyst Layer Electrolyte Resistance in PEFCs Using Electrochemical Impedance Spectroscopy. J. Electrochem. Soc..

[37] Wang C., Chen X., Xiang X., Zhang H., Huang Z., Huang X., Zhan Z. (2023). Study on Self-Humidification in PEMFC with Crossed Flow Channels and an Ultra-Thin Membrane. Polymers.

[38] Hao D., Shen J., Hou Y., Zhou Y., Wang H. (2016). An Improved Empirical Fuel Cell Polarization Curve Model Based on Review Analysis. Int. J. Chem. Eng..

[39] Zhang J., Tang Y., Song C., Xia Z., Li H., Wang H., Zhang J. (2008). PEM fuel cell relative humidity (RH) and its effect on performance at high temperatures. Electrochim. Acta.

[40] Butori M., Eriksson B., Nikolić N., Lagergren C., Lindbergh G., Lindström R.W. (2023). The effect of oxygen partial pressure and humidification in proton exchange membrane fuel cells at intermediate temperature (80–120 °C). J. Power Sources.

[41] Kim J., Lee S.M., Srinivasan S., Chamberlin C.E. (1995). Modeling of Proton Exchange Membrane Fuel Cell Performance with an Empirical Equation. J. Electrochem. Soc..

