# Supplementary material for: Experimental and Simulation Study of Proton Exchange Membrane Fuel Cell with 12 µm Thick Membrane over the Temperature Range of 80 °C to 120 °C
Source: Membranes (Basel). 2025 Mar 1;15(3):72. doi: 10.3390/membranes15030072 (PMC11943660; doi:10.3390/membranes15030072)
Supplement: Supplementary file 1 [file membranes-15-00072-s001.zip › membranes-3479253-supplementary.pdf]

Experimental and Simulation Study of Proton Exchange Membrane Fuel Cell with 12 μm thick membrane over the Temperature Range of 80 °C to 120 °C

Yunfei Zhang [1,2], Zhengrui Xiao [1,2], Xiaoyang Zhao [1,2], Jian Wang [1,2], Yadong Wang [1,2] and Jun Yu [1,2,*]

[1] *State Key Laboratory* of Advanced Technology for Materials Synthesis and Processing, Wuhan university of Technology, Wuhan 430070, China

[2] [1] *Hubei Key Laboratory of Fuel Cells, Wuhan 430070, China*

[*] Email: yujun@whut.edu.cn

Supplementary information

ECSA calculation

Electrochemical surface area (ECSA) values were calculated by integrating the area under part of CV curves. The relevant formula (eq (S1)) is expressed by:

$$\mathrm{ECSA} = \frac{0.1\times S}{0.21\times v\times m_{\mathrm{pt}}} \tag{S1}$$

S represents the hydrogen adsorption area (mC·cm^{-2}) obtained by integrating the CV curve above the current density value corresponding to 0.4 V, v is the CV scanning rate (mV·s^{-1}), and m_{pt} is the platinum loading on the catalyst layer (mg·cm^{-2}).

Polarization curve fitting

The output voltage V of the fuel cell is expressed as follows [1],

$$V = E_{\mathrm{rev}} - V_{\mathrm{overpotential}} = E_{\mathrm{rev}} - \eta_{\mathrm{act}} - \eta_{\mathrm{ohm}} - \eta_{\mathrm{con}} \tag{S2}$$

where E_{rev} is the reversible overpotential, also known as ideal thermodynamic voltage, [2] E_{rev} as shown in eq (S3),

$$E_{\mathrm{rev}} = 1.23 - 0.85\times 10^{-3}\times (T - 298.15) + \frac{RT}{2F}\times\left(\ln P_{\mathrm{H_2}} + \frac{1}{2}\ln P_{\mathrm{O_2}} - \ln P_{\mathrm{H_2O}}\right) \tag{S3}$$

Where F is the Faraday constant (96485 C·mol^{-1}), and T is the temperature in Kelvin (K), R is the ideal gas constant (8.314 J·mol^{-1}·K^{-1}), $P_{\mathrm{H_2}}$, $P_{\mathrm{O_2}}$ and $P_{\mathrm{H_2O}}$ are the partial pressures of hydrogen, oxygen and water vapor inside the cell respectively, with the unit of atm.

The partial pressures of various substances are as follows: [3,4]

$$P_{\mathrm{H_2O}} = RH \times P_{\mathrm{sat}} \tag{S4}$$

RH is the relative humidity of the cathode or anode, P_{sat} is the water saturation vapor pressure related to temperature. The partial pressure of hydrogen $P_{\mathrm{H_2}}$ at the anode and the partial pressure of oxygen $P_{\mathrm{O_2}}$ at the cathode can be calculated as follows:

$$P_{H_2} = P_{an} - P_{H_2O} \tag{S5}$$

$$P_{O_2} = 0.21 \times \left(P_{ca} - P_{H_2O}\right) \tag{S6}$$

All the relevant pressures mentioned above are absolute pressures. P_{an}, P_{ca} are the total pressures of the anode and cathode respectively. According to eq (S3)-(S6), the E_{rev} and partial pressure of reactants involved in the experiment are calculated as shown in Table S2.

Ohmic overpotential η_{ohm} displays a linear variation with current density i, which can be estimated by the eq (S7): [5]

$$\eta_{\text{ohm}} = iR_{\text{ohm}} \tag{S7}$$

R_{ohm} ($\Omega\cdot cm^{-2}$) represents the ohmic resistance predominantly contributed by the PEM.

The DC voltage and R_{ohm} in the low current density range were measured at 6, 8, 10, 40 and 100 $mA\cdot cm^{-2}$, and the η_{ohm} was calculated according to eq (S7). Since the consumption of oxygen and the generation of water are relatively low at low current density (2-100$mA\cdot cm^{-2}$), the influence of concentration overpotential η_{con} can be neglected. Therefore, at low current density range, equation (S2) is transformed into eq (S8):

$$\eta_{\text{act}} = E_{\text{rev}} - V - \eta_{\text{ohm}} \tag{S8}$$

E_{rev}, V, and η_{ohm} can be obtained by polarization curve and EIS tests. At this time, parameters a and b were obtained by linear fitting combined with eq (S8) and Tafel equation shown in eq (S9). Therefore, the η_{act} across the entire current density range was calculated using the derived Tafel equation. [1]

$$\eta_{\text{act}} = a + b\log(i) \tag{S9}$$

where a is the fitting constant, and b is called the Tafel slope ($mV\cdot dec^{-1}$), i is current density ($mA\cdot cm^{-2}$).

An empirical correction [1] used to estimate the η_{con} by eq (S10):

$$\eta_{\mathrm{con}} = me^{(ni)-1} \quad \text{(S10)}$$

Both m and n are fitting parameters. In this study, after obtaining η_{ohm} and η_{act} through eq (S7) and eq (S9), η_{con} can be obtained by subtracting them from the total voltage loss $V_{\mathrm{overpotential}}$.

Table S1. Physical parameters and boundary conditions for modeling. [6]

Parameter	Value
Temperature (K)	353.15/373.15/393.15
Anode/cathode outlet pressure (kPa)	200/250/300 (abs)
Thickness of PEM (mm)	0.012/0.050
Width of PEM; CL; GDL (mm)	50
Channel width; depth (mm)	0.83; 0.83
Porosities of GDB; MPL; CL	0.78; 0.6; 0.78
Absolute permeabilities of GDB; MPL; CL (m^2)	8×10^{-12}; 5×10^{-13}; 3×10^{-14}
Contact angel of GDB; MPL; CL (°)	130; 140; 120
Specific heat capacities of GDB; MPL; CL; PEM ($J\cdot kg^{-1}\cdot K^{-1}$)	710; 710; 3300; 2000
Thermal conductivities of GDB; MPL; CL; BP ($W\cdot m^{-1}\cdot K^{-1}$)	1.7; 1.7; 8; 85.5
Electrical conductivities of CL; GDB; MPL; BP ($S\cdot m^{-1}$)	1000; 5000; 5000; 83000

Table S2. Partial pressures and equilibrium overpotentials under different conditions.

		80 ℃			100 ℃			120 ℃		
43%RH	p_{an}/p_{ca} (kPa)	200	250	300	200	250	300	200	250	300
	p_{H_2O} (atm)	0.20			0.43			0.85		
	p_{H_2} (atm)	1.79	2.28	2.78	1.56	2.04	2.54	1.14	1.63	2.13
	p_{O_2} (atm)	0.38	0.48	0.58	0.33	0.43	0.54	0.24	0.34	0.45
	E_{rev} (V)	1.209	1.215	1.219	1.178	1.185	1.190	1.142	1.151	1.158
30%RH	p_{H_2O} (atm)	0.14			0.30			0.59		
	p_{H_2} (atm)	1.85	2.34	2.84	1.69	2.17	2.67	1.40	1.89	2.38
	p_{O_2} (atm)	0.39	0.49	0.60	0.35	0.46	0.56	0.29	0.40	0.50
	E_{rev} (V)	1.215	1.221	1.225	1.186	1.192	1.197	1.154	1.161	1.167

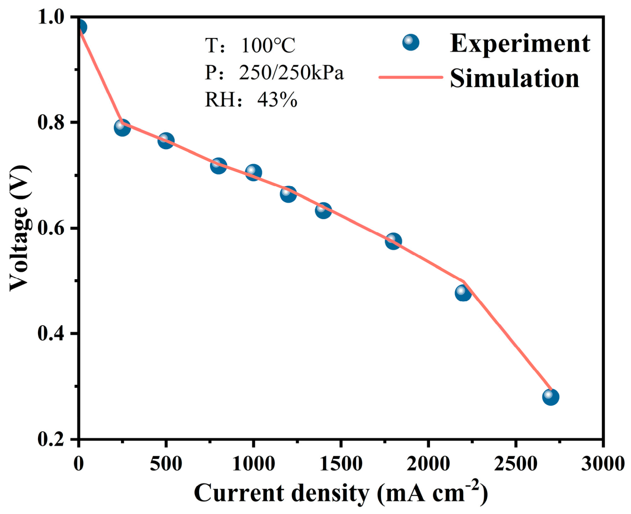

Figure S1. Polarization curve by experimental and modeling

REFERENCES

1. Hao, D.; Shen, J.; Hou, Y.; Zhou, Y.; Wang, H. An Improved Empirical Fuel Cell Polarization Curve Model Based on Review Analysis. *Int. J. Chem. Eng.* **2016**, *2016*, 1-10. DOI: 10.1155/2016/4109204
2. Yuan, X.; Wang, H. PEM Fuel Cell Fundamentals. In *PEM Fuel Cell Electrocatalysts and Catalyst Layers: Fundamentals and Applications*, Zhang, J. (Ed.), Springer London, London, **2008**, pp. 1-87. DOI: 10.1007/978-1-84800-936-3_1
3. Zhang, J.; Tang, Y.; Song, C.; Xia, Z.; Li, H.; Wang, H.; Zhang, J. PEM fuel cell relative humidity (RH) and its effect on performance at high temperatures. *Electrochim. Acta* **2008**, *53*, 5315-5321. DOI: 10.1016/j.electacta.2008.02.074
4. Butori, M.; Eriksson, B.; Nikolić, N.; Lagergren, C.; Lindbergh, G.; Lindström, R.W. The effect of oxygen partial pressure and humidification in proton exchange membrane fuel cells at intermediate temperature (80 – 120℃). *J. Power Sources* **2023**, *563*. DOI: 10.1016/j.jpowsour.2023.232803
5. Kim, J.; Lee, S.M.; Srinivasan, S.; Chamberlin, C.E. Modeling of Proton Exchange Membrane Fuel Cell Performance with an Empirical Equation. *J. Electrochem. Soc.* **1995**, *142*, 2670. DOI: 10.1149/1.2050072
6. Wang, C.; Chen, X.; Xiang, X.; Zhang, H.; Huang, Z.; Huang, X.; Zhan, Z. Study on Self-Humidification in PEMFC with Crossed Flow Channels and an Ultra-Thin Membrane. *Polymers* **2023**, *15*, 4589. DOI: 10.3390/polym15234589
